# Dietary lactoferrin has differential effects on gut microbiota in young versus middle-aged APPswe/PS1dE9 transgenic mice but no effects on cognitive function

**DOI:** 10.29219/fnr.v65.5496

**Published:** 2021-10-18

**Authors:** Huan-Huan Zhou, Guiping Wang, Lan Luo, Wei Ding, Jia-Ying Xu, Zengli Yu, Li-Qiang Qin, Zhongxiao Wan

**Affiliations:** 1 Department of Nutrition and Food Hygiene, School of Public Health, Soochow University, Suzhou, China; 2 School of Physical Education, Soochow University, Suzhou, China; 3 Laboratory Animal Center, Medical College of Soochow University, Suzhou, China; 4 School of Radiation Medicine and Protection, Soochow University, Suzhou, China; 5 School of Public Health, Zhengzhou University, Zhengzhou, China

**Keywords:** Alzheimer’s disease, lactoferrin, cognitive function, gut microbiota, amyloid β

## Abstract

**Background:**

Existing evidence suggest that lactoferrin might be beneficial for Alzheimer’s disease, while precise mechanisms are not fully elucidated.

**Objective:**

To determine the effects of lactoferrin intervention on cognitive function from APPswe/PS1dE9 (APP/PS1) mice, and potential mechanisms involved.

**Design:**

Both the young and middle-aged male APP/PS1 mice were divided into the control and lactoferrin intervention groups with 16 weeks’ intervention.

**Results:**

Lactoferrin had no effects on cognitive function for both the young and middle-aged mice, and no key markers involved in Aβ, tau pathology, neuro-inflammation and synaptic plasticity were altered after lactoferrin intervention. With regards to gut microbiota profiles, in the young APP/PS1 mice, lactoferrin elevated the α diversity index including ACE and Chao 1, and reduced the relative abundance of the genera *Bacteroides* and *Alistipes* and elevated *Oscillibacter*; in addition, *Oscillibacter, Anaerotruncus, EF096579_g, EU454405_g, Mollicutes_RF39, EU474361_g, EU774448_g, and EF096976_g* were specifically abundant via linear discriminant analysis with effect size (LEfSe) analysis. In the middle-aged APP/PS1 mice, the relative abundance of the phylum *Proteobacteria*, as well as the genera *Oscillospira, Coprococcus*, and *Ruminococcus* was significantly reduced post lactoferrin; additionally, *S24_7*, *Bacteroidia*, *Bacteroidetes,* and *Methylobacterium* were specific via LEfSe analysis in the lactoferrin group.

**Conclusions:**

Dietary lactoferrin might be beneficial for gut microbiota homeostasis although it might have no effects on cognition.

## Popular scientific summary

- Lactoferrin (Lf) elevated ACE and Chao 1 from young APP/PS1 mice. Lf reduced *Bacteroides* and *Alistipes* and elevated *Oscillibacter* from young mice. Lf reduced phylum *Proteobacteria* from middle-aged mice.

Lactotransferrin or lactoferrin (Lf), is a multifunctional, non-heme iron-binding glycoprotein, which belongs to the transferrin family (1). Lf has been reported to have multiple biological functions such as immuno-modulatory effects (2), antioxidant effects (3) and anticancer activities (4). Lf primarily exists in two forms, that is, Fe^3+^ free/associated (apo-Lf) and Fe^3+^ saturated (holo-Lf) forms (5). Over the past two decades, increasing evidence at both the animal and population levels suggests that Lf intervention might be protective against Alzheimer’s disease (AD). To be specific, as early as in 1999, Fillebeen et al. (6) reported that Lf was capable of crossing the blood–brain barrier via receptor-mediated transcytosis. A recent open-label, randomized, controlled pilot study conducted in AD patients demonstrated that Lf administration (250 mg/d) for a total of 3 months could significantly improve cognitive function (7); this might be associated with its effects on the Akt/PTEN pathway, consequently affecting key inflammatory and oxidative stress players involved in AD pathology. Similarly, intranasal human Lf (hLf) administration in APPSwe/PS1dE9 (APP/PS1) mice improved cognitive function (8). However, there is also evidence indicating that Lf might contribute to AD pathology. For example, the presence of Lf had been detected in senile plaques and neurofibrillary tangles (NFTs) in the limbic system of APP transgenic mice (9), as well as in AD patients (10). Additionally, some researchers postulated that the presence of Lf in AD could be a counter-regulatory defense mechanism to fight against the inflammatory cascade that normally existed in AD (11). Nevertheless, the direct role of Lf in AD requires further exploration considering the heavy burden of AD and no effective therapy at this time point (12).

The role of gut microbiota in the development of AD has been greatly appreciated in recent years (13). Directly, gut microbiota could secrete quantities of amyloids and lipopolysaccharides, which might contribute to AD pathology such as neuro-inflammation and Aβ plaques (14). Indirectly, imbalanced gut microbiota profiles are also associated with inflammation, obesity, and type 2 diabetes (15), all of which are risk factors for AD. In the meantime, accumulating evidence confirmed that both human and bovine derived lactoferrin or bovine lactoferrin-derived lactoferricin (Lfcin) B are capable of modulating the fecal microbiome in different species such as in very low birth weight infants (16), suckling piglets (17), and enterohemorrhagic *Escherichia coli* (EHEC) O157:H7 mouse model (18). However, it remains unclear whether altered gut microbiome post Lf intervention might contribute to Lf’s protective effects against AD.

Advanced age *per se* is one of the main risk factors for AD; age-related changes such as altered composition of the gut microbiota may be potentially involved in the onset of AD (13). APP/PS1 transgenic mice, which demonstrate age-dependent cognitive deficits, have been extensively used for AD research. At 4 months of age, these mice firstly develop Aβ plaques following other AD pathologies including activated microglia and astrocytes surrounding Aβ plaques and phosphorylation of Tau (19). Recent findings also suggest that age-related alterations including but not limited to hippocampal AD pathology (20) and microbiota diversity (21) existed in this AD mouse model. Therefore, it is likely that the beneficial effects of Lf on cognitive function in APP/PS1 mice might be age-dependent, and altered gut microbiota profiles, as well as AD pathology might be responsible for age-related improvement in cognitive function post Lf intervention. Consequently, we aimed to determine the effects of Lf intervention on cognitive function by utilizing both young (10 weeks) and middle-aged (24 weeks) APP/PS1 mice as models; we also investigated alterations of the key makers involved in AD pathology (i.e. Aβ, tau phosphorylation, neuro-inflammation and synaptic plasticity related proteins), as well as the cecal microbiome post Lf intervention.

## Materials and methods

### Materials

AIN93-G standard and modified diet was purchased from Trophic Animal Feed High-Tech Company, Ltd. (Nantong, China). Lactoferrin of milk origin was purchased from Hilmar Cheese Company (CA, USA). The reagents, as well as molecular weight marker and nitrocellulose membranes for sodium dodecyl sulphate–polyacrylamide gel electrophoresis (SDS-PAGE) were purchased from Beyotime Institute of Technology (Jiangsu, China) and Bio-Rad (CA, USA), respectively. The antibodies were obtained from ImmunoWay Biotechnology Company (DE, USA) as follows: phosphorylation of Tau at serine 396 (p-Tau serine396) (YP0263), p-Tau serine404 (YP0264). Anti-post synaptic density protein-95 (PSD95) (AJ1661a) was from Abgent (SD, USA). Antibodies directly against β-site APP cleaving enzyme 1 (BACE1) (5606), synaptophysin (5461), glial fibrillary acidic protein (GFAP) (3670) ,and β-Actin (4970) were from Cell Signaling (MA, USA). These following antibodies were obtained from Abcam (Shanghai, China): insulin degrading enzyme (IDE) (ab32216), cathepsin B (ab58802), and brain derived neurotrophic factor (BDNF) (ab108383). An antibody against ionized calcium binding adaptor molecule 1 (Ibα1) (016-20001) was from Wako (Osaka, Japan).

### Animals and intervention

All male APP/PS1 transgenic mice (B6C3F1 background, APPswe strain, cleanliness of SPF) were purchased from Nanjing Model Animal Center (Nanjing, China). All animal procedures followed the Guidelines in the Care and Use of Animals and were approved by the Soochow University Animal Welfare Committee (approval no. 201809A358). All mice were raised in standard plastic cages under specific pathogen-free conditions under conditions of appropriate temperature (20–25°C), humidity (55–60%), and a 12-h light–dark cycle. For experiment 1 in young APP/PS1 mice, after 1 week of acclimatization, a total of 14 male APP/PS1 mice (10-week-old) were randomly assigned into two groups with either a standard AIN-93G diet served as control group (Young Tg control group, YTGCon) or AIN-93G diet supplemented with 0.8% lactoferrin (i.e. 800 mg lactoferrin dissolved into 100 g AIN-93G diet) (YTGLf), 7 mice *per* group. Similarly, for experiment 2 in middle-aged APP/PS1 mice, the 24-week-old male APP/PS1 transgenic mice (*N* = 14) were divided into a middle-aged control group (ATGCon) and a middle-aged lactoferrin diet group (ATGLf), respectively, with the same diet intervention as described above. The total intervention duration was 16 weeks for both the young and middle-aged mice. Over the intervention periods, the average daily dietary intake *per* mice was roughly 4 g/day; thus the average daily Lf intake was about 32 mg/d. The selection of the above dosage for Lf was based on findings from our (22, 23) and other (24) research groups. Water and specific food were allowed for mice *ad libitum*. Body weight and food intake were measured on a weekly basis over the duration of the study.

### Glucose and insulin tolerance test

At the end of the intervention, intraperitoneal glucose and insulin tolerance test (ITT) were performed as described previously by our laboratory (25). Briefly, mice were intraperitoneally (I.P.) injected with glucose (1.5 g/kg body weight) during the glucose tolerance test (GTT) in the morning (8 a.m.) after a total of 6 h fasting. The next day, mice were given an I.P. injection of insulin (0.5 IU/kg body weight) in the morning (8 a.m.) without fasting. The blood glucose level was determined by tail vein sampling at the following time points (i.e. 0, 15, 30, 45, 60, 90, and 120 min post injection) via a hand-held glucometer. Changes in glucose over time were plotted and the total area under the curve (AUC) of glucose levels was calculated.

### Morris water maze test

The Morris water maze (MWM) test was used to evaluate the spatial learning and memory ability of mice as described previously by our laboratory with small modifications (26). Briefly, the test consists of 5 consecutive days of navigation trial and a 1 day of probe trial. During the navigation trial, mice were allowed to find the platform within 80 s; those who had found the platform within 80 s were allowed to rest for 10s, otherwise they would be forced to stay at the platform for 20 s. The time that the mice required for reaching the platform (escape latency) was recorded to assess spatial learning ability. During the probe trial, the platform was removed to assess the retention of tasks of the mice. The number of mice crossing the platform area, the swimming distance, and time spent in the targeted quadrant were recorded to evaluate the spatial memory capacity. Supermaze tracking software (Shanghai Xinsoft Information Technology Co., Ltd., Shanghai, China) was used for data collection and analysis.

### Sample collection and preservation

After behavioral test and overnight fasting, mice were sacrificed with the brains immediately removed thereafter. The hippocampus and parietal-temporal cortex of the brain were separated and frozen rapidly in liquid nitrogen and stored at −80°C for further analysis. Cecal contents (150–200 mg) were also harvested and snap frozen in liquid nitrogen and then stored at −80°C for further 16S rRNA Gene Sequencing and Microbiota Analysis.

### Western blotting

Western blotting was used to determine proteins of interest from both the hippocampus and parietal cortex as described previously by our laboratory (26). In short, a total of 20 μL samples were separated by SDS-PAGE gel electrophoresis, and then transferred to a nitrocellulose membrane using a Trans-blot Turbo Transfer System (Bio-Rad). Membranes were blocked in 5% milk powder solution, and then incubated with respective primary antibodies rocking slowly overnight in the fridge. The second day, the membranes were incubated with proper secondary antibodies at room temperature for 1 h, and imaged by the Syngene chemi-imaging system (MD, USA) using Immobilon western chemiluminescent horseradish peroxidase (HRP) substrate. Beta-Actin was used as the internal control for normalization.

### 16S rRNA gene sequencing and microbiota analysis

DNA was extracted by using the PowerMax extraction kit (MoBioLaboratories, CA, USA) and stored at −20°C for standby. The quantity and quality of DNA were determined by using the NanoDrop ND-1000 spectrophotometer (Thermo Fisher Science, MA, USA). The polymerase chain reaction (PCR) system was utilized to amplify the V4 region of the bacteria’s 16S ribosomal RNA (rRNA) gene. The PCR reactions were performed according to the following program: 30 s of pre-denaturation at 98°C, 30 cycles of 15 s for denaturation at 98°C, 15 s for annealing at 58°C, and 15 s for elongation at 72°C, and a final extension at 72°C for 1 min. The resulted PCR products were further purified using AMPure XP Beads (Beckman Coulter, IN, USA) and quantified using the PicoGreends DNA Assay Kit (Invitrogen, CA, USA). The Illlumina HiSeq 4,000 pair-end 2×150 BP platform was used for quantitative sequencing. The data of each sample was separated from the original data according to the Barcode sequence and primer sequence. After truncating Barcode and primer sequence, Vsearch v2.4.4 was used to splice the reads of each sample to obtain raw tags. Then through a series of preprocessing, including the removal of low-quality bases, ambiguous bases and adapter sequences and the detection of chimeric tags, clean tags were obtained. On the basis of sequence identification, clean tags were clustered into operational taxonomic unit (OTU) at 97% confidence threshold. Species annotation of the representative sequence was carried out through VSEARCH based on SILVA128 database (27). QIIME software was used to calculate the alpha diversity index of the OTU level, including Chao1, ACE, Shannon Index, and Simpson index, and the significance was determined by using Student’s *t* test within the same age group. Beta (β) diversity of the microbial community structure of different samples was analyzed by Principal Coordinate Analysis (PCoA) based on UniFrac distance measurement (28). Linear discriminant analysis (LDA) with effect size (LEfSe) was performed to predict biomarkers specifically abundant in each group. The cut off value was set as the absolute LDA score (log10) >2.0 (29).

## Statistical analysis

All data are presented as mean ± SEM. Except for data of gut microbiota, a Student’s *t* test was used for comparisons between YTGCon and YTGLf groups, as well as between ATGCon and ATGLf groups because we conducted the two experiments separately. Statistical significance was established at *P* < 0.05.

## Results

### Glucose and insulin tolerance test

**Fig. 1:**
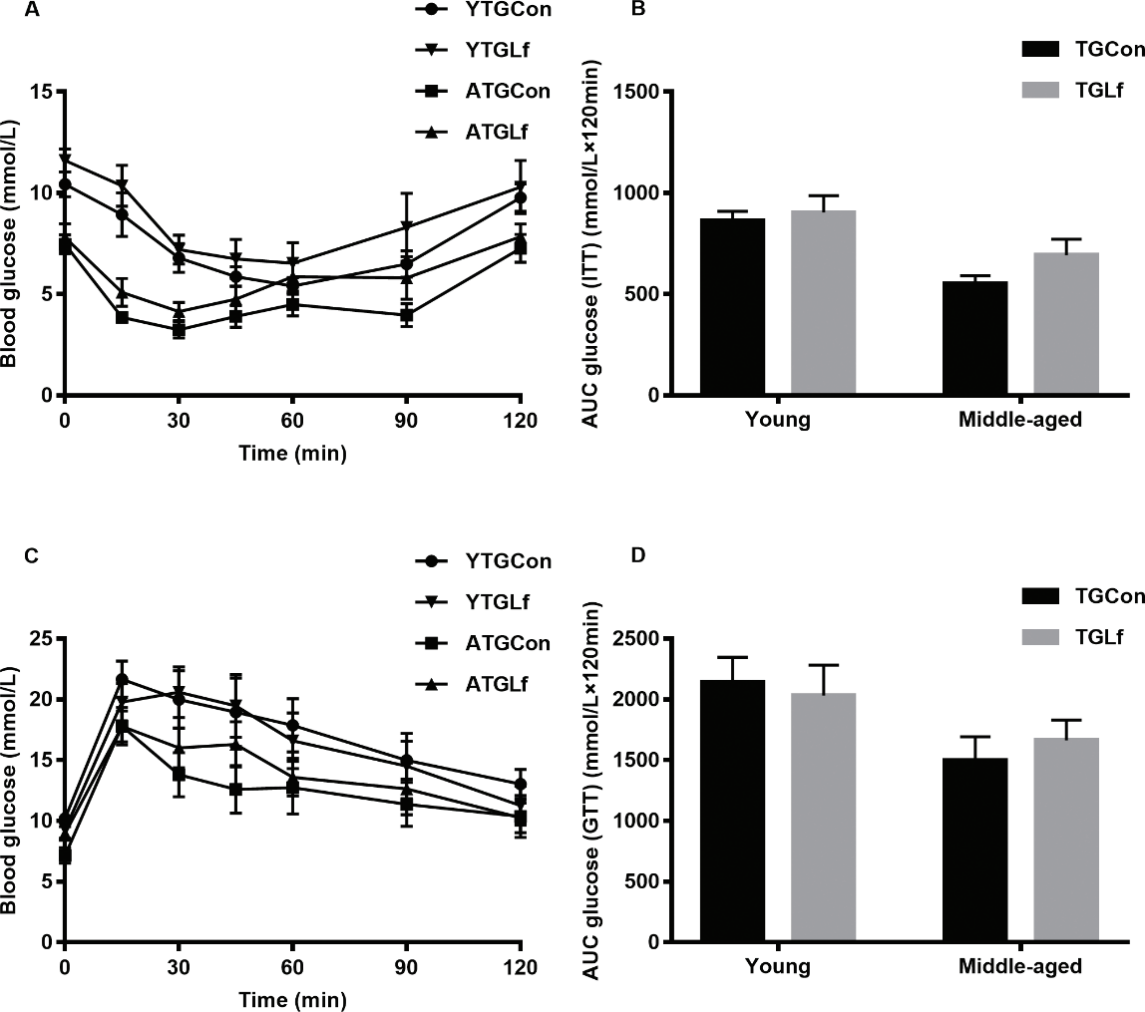
Glucose and Insulin Tolerance Test. Glucose levels (a) at 15 min, 30 min, 45 min, 60 min, 90 min, 120 min, and the total area under the curve (AUC) (b) during ITT. Glucose levels (c) at different timepoints and AUC (d).

There was no significant difference for glucose levels at the detected timepoint, as well as no difference for incremental AUC between groups within the same age for both ITT (Fig. 1a, b) and GTT tests (Fig. 1c,d). Overall, our results suggested that lactoferrin intervention in our study might have no effect on glucose and insulin tolerance in both the young and middle-aged APP/PS1 mice.

### Behavioral performance via MWM

**Fig. 2:**
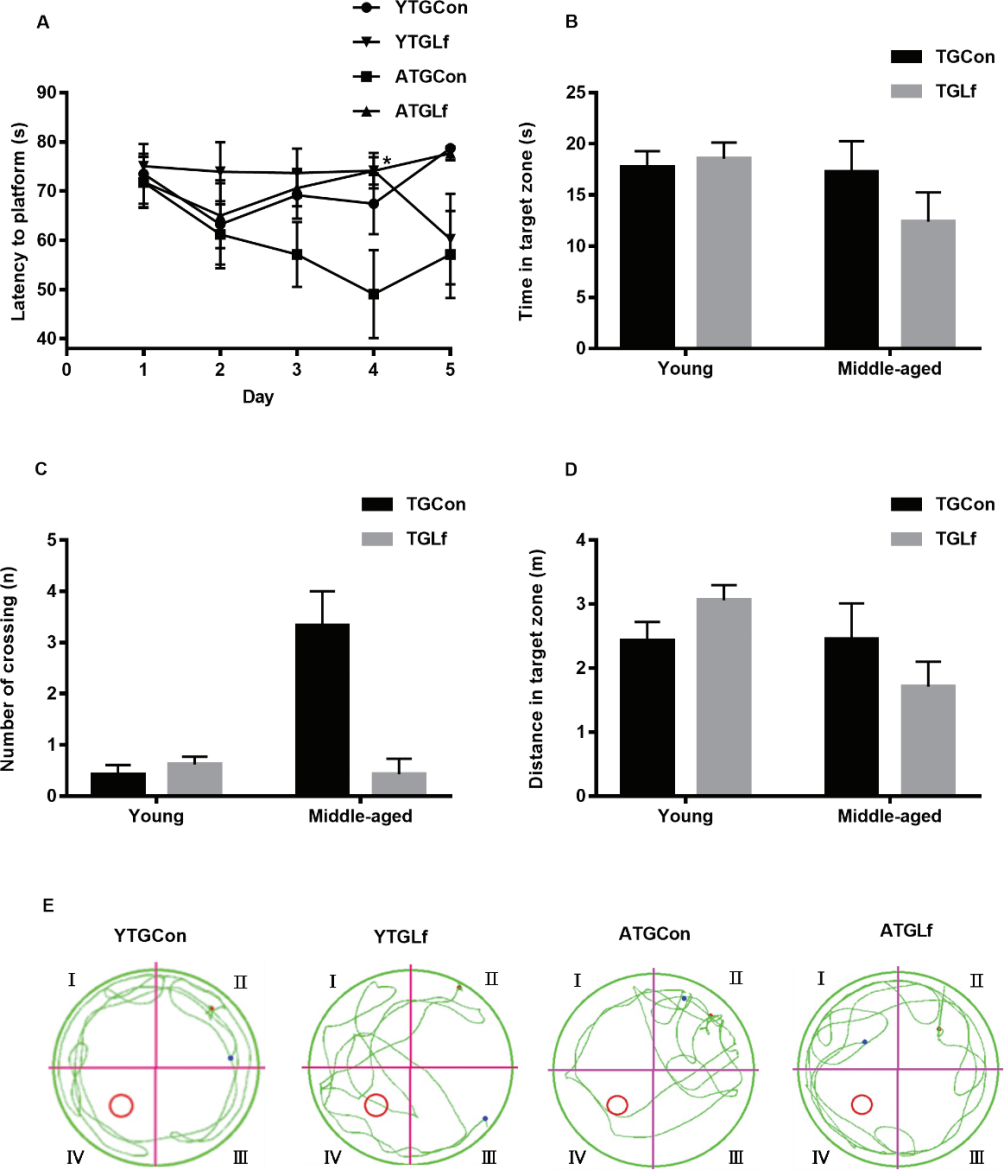
Behavioral Performance via MWM. (a) Mean escape latency to hidden platform on 5 days of navigation test. During probe trails, time spent in the target quadrant (b), number of crossing the previous hidden platform (c), and swimming distance in the target quadrant (d) were recorded. (e) Representative motion tracking of the mice in the different groups. All values were presented as means ± SEM.

As shown in Fig. 2a, on the fourth day of the navigation trial test, mice from the ATGLf group had elevated escape latency compared to the ATGCon group, while there was no difference for escape latency between YTGCon and YTGLf groups on all the test days of the navigation trial. During the probe trial test, there was no significant difference for time in the target zone, number of crossing and distance in the target zone between TGCon and TGLf groups within the same age (Fig. 2b–d). The swimming paths of representative mice in each group were shown in Fig. 2e. Collectively, our results suggested that 16-weeks’ Lf intervention might have no effect on the spatial learning and memory abilities of both the young and middle-aged APP/PS1 mice, at least via MWM.

### Proteins involved in A β metabolism and phosphorylation of tau protein

**Fig. 3:**
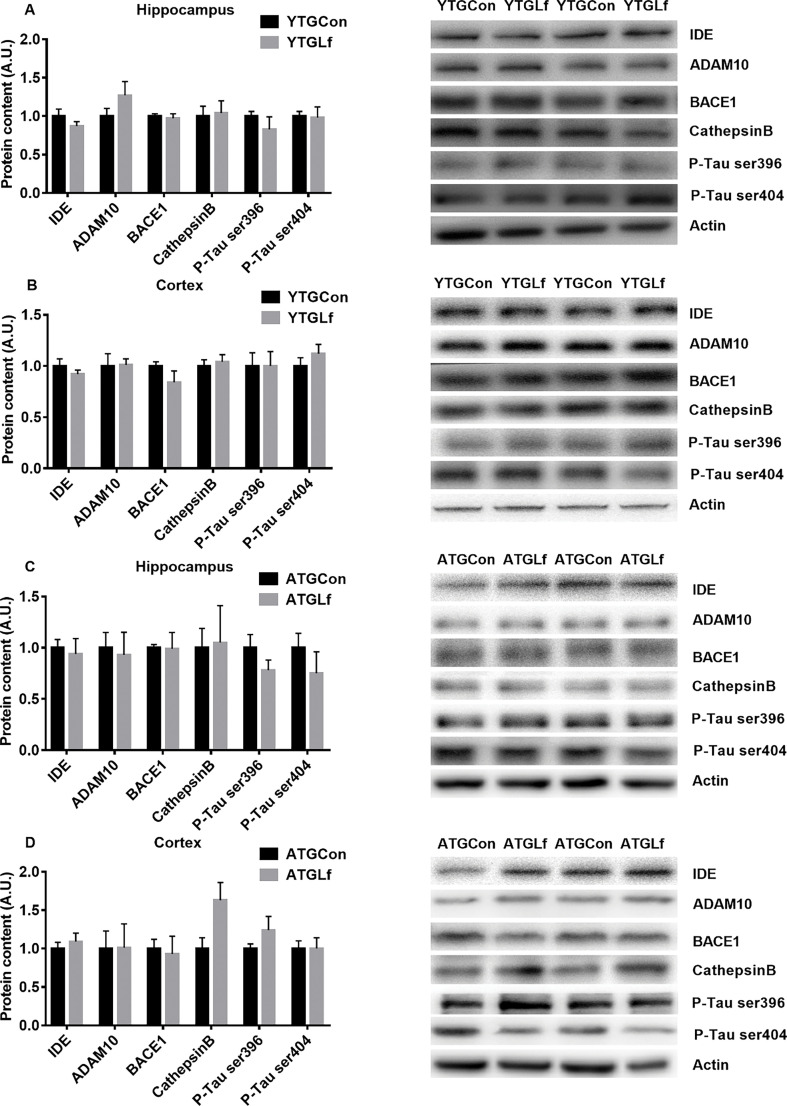
Aβ aggregation and phosphorylation of Tau Associated Protein Expression in young APP/PS1 mice, protein expression of IDE, ADAM10, BACE1, Cathepsin B, P-Tau ser396, and P-Tau ser404 in the hippocampus (a) and cortex (b) were detected by western blotting. In middle-aged APP/PS1 mice, the above proteins were shown in the hippocampus (c) and cortex (d) via western blotting. All values were presented as mean + SEM. A.U. means arbitrary units. Representative blots were shown in the right panel in a–d.

We further examined the protein expression of key indicators involved in Aβ pathology including IDE, a disintegrin and metalloproteinase 10 (ADAM10), BACE1 and cathepsin B, as well as p-Tau at ser396 and ser404. No significant differences for IDE, ADAM10, BACE1, cathepsin B, p-Tau ser396, and p-Tau ser404 protein expression were observed from both the hippocampus and cortex between TGCon and TGLf groups for both the young and middle-aged APP/PS1 mice (Fig. 3a–d).

### Proteins involved in neuro-inflammation and synaptic plasticity

**Fig. 4:**
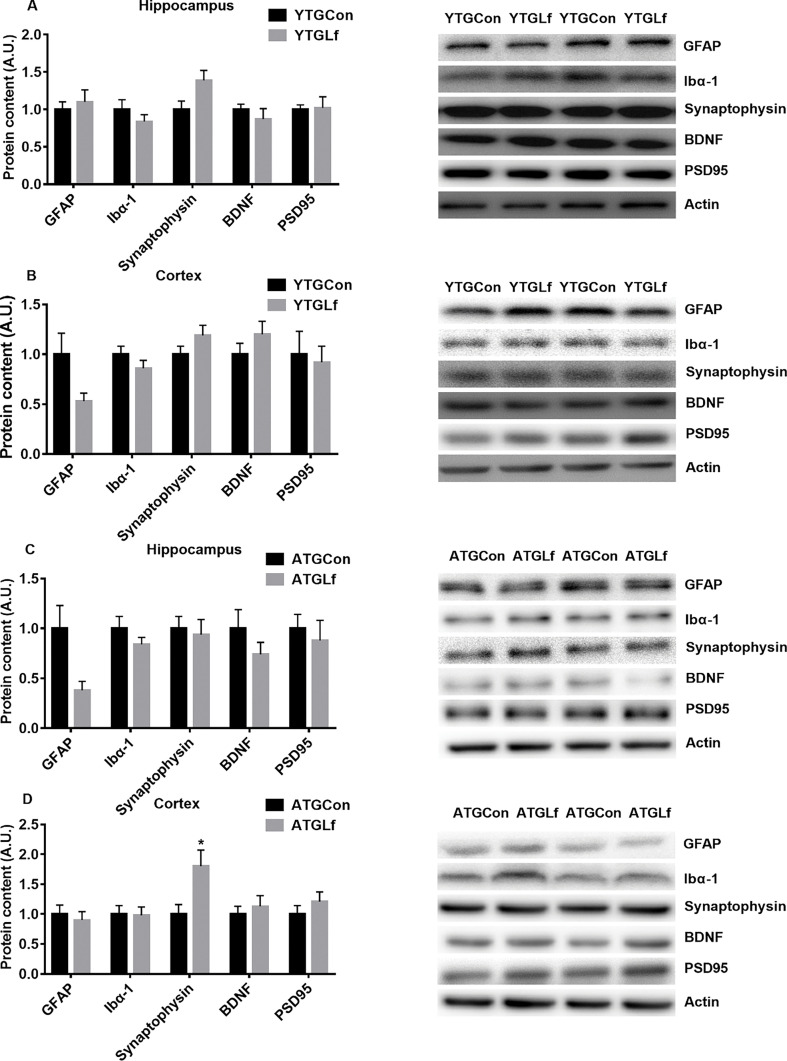
Neuro-inflammation Related Markers and Synaptic Plasticity in young APP/PS1 mice, protein expression of GFAP, Ibα1, synaptophysin, BDNF, PSD95 in the hippocampus (a) and cortex (b) were detected by western blotting. In middle-aged APP/PS1 mice, the above proteins were shown in the hippocampus (c) and cortex (d) via western blotting. All values were presented as mean + SEM. A.U. means arbitrary units. Representative blots were shown in the right panel in a–d.

For the young APP/PS1 mice, there was no significant difference for GFAP, Ibα1, synaptophysin, BDNF, and PSD95 protein expression in both the hippocampus and cortex between YTGCon and YTGLf groups (Fig. 4a, b). For the middle-aged APP/PS1 mice, a significant elevation in synaptophysin protein expression was observed in the cortex for the ATGLf group relative to ATGCon, while there was no significant difference for GFAP, Ibα1, BDNF, and PSD95 protein expression in both the hippocampus and cortex, as well as synaptophysin in hippocampus between ATGCon and ATGLf groups (Fig. 4c, d).

### Compositions and overall structure of gut microbiota

**Fig. 5:**
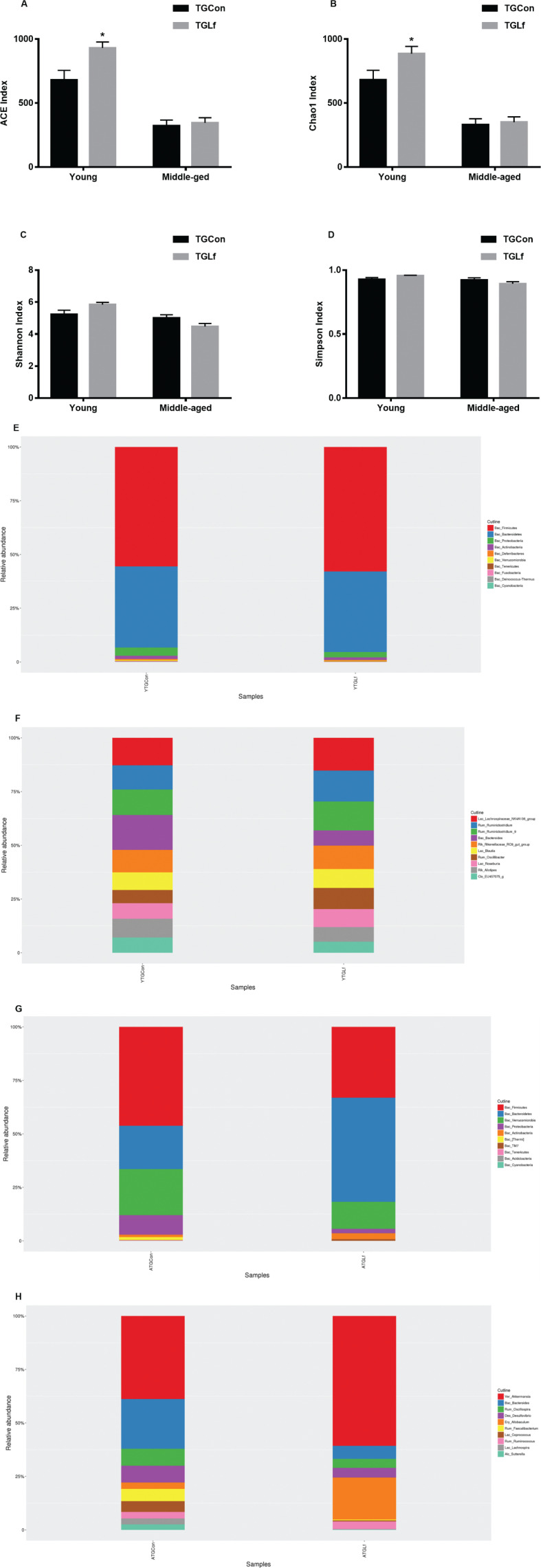
Alterations in Gut Microbiota Composition. The alpha diversity of the ACE index (a), Chao1 index (b), Shannon index (c), Simpson index (d) displays the microbial diversity of each group. Relative abundance of top 10 microbes in young APP/PS1 mice at the rank of phylum (e) and genus (f), and in middle-age APP/PS1 mice at the rank of phylum (g) and genus (h) was shown.

An average of 1,26,425 clean tags were obtained from cecal samples of the young APP/PS1 mice, and 90,002 clean tags were obtained from cecal samples of the middle-aged APP/PS1 mice. The ACE and Chao1 indexes were significantly increased in the YTGLf group compared to the YTGCon group (Fig. 5a, b). However, the Shannon and Simpson index showed no significant difference between groups within the same age (Fig. 5c, d). Based on the taxonomic analysis of OTU representative sequences, the distributions of the top 10 bacteria at phylum and genus level in young and middle-aged mice were shown in Fig. 5e–h, respectively. For the young APP/PS1 mice, *Firmicutes*, *Bacteroidetes*, and *Proteobacteria* were the dominant bacteria at the phylum level (Fig. 5e), and *Lachnospiraceae_NK4A136_group*, *Ruminiclostridium*, and *Ruminiclostridium_9* dominated the microbiota at the genus level (Fig. 5f). For the middle-aged APP/PS1 mice, the top three bacteria at the phylum level were *Firmicutes*, *Bacteroidetes*, and *Verrucomicrobia* (Fig. 5g); at the genus level were *Akkermansia*, *Bacteroides,* and *Oscillospira* (Fig. 5h). We further analyzed the difference of gut microbiota at both phylum and genus levels between each two groups within the same age by using the Mann–Whitney U test. As shown in Table 1, for the young APP/PS1 mice, there was no significant difference noted for the top 10 bacteria between YTGCon and YTGLf groups at the phylum level; while at the genus level, the relative abundance of *Bacteroides* and *Alistipes* of YTGLf group was lower than that of YTGCon group, and the relative abundance of *Oscillibacter* in the YTGLf group was higher than that of YTGCon group. For the middle-aged APP/PS1 mice, the relative abundance of the phylum *Proteobacteria*, as well as *Oscillospira, Coprococcus,* and *Ruminococcus* at the genus level from the ATGLf group was significantly reduced compared to the ATGCon group.

**Table 1:** The relative abundance of the top 10 gut bacterial genera at the phylum and genus level (%) from both young and middle-aged APP/PS1 mice

Level	YTGCon	YTGLf	Level	ATGCon	ATGLf
Phylum			Phylum		
Firmicutes	55.43 ± 4.00	57.74 ± 4.18	Firmicutes	17.19 ± 5.70	8.30 ± 1.64
Bacteroidetes	37.62 ± 4.77	37.37 ± 4.17	Bacteroidetes	7.54 ± 5.72	12.21 ± 4.69
Proteobacteria	3.80 ± 0.66	2.49 ± 0.18	Verrucomicrobia	8.00 ± 5.47	3.17 ± 2.60
Actinobacteria	1.48 ± 0.24	1.17 ± 0.23	Proteobacteria	3.42 ± 1.69	0.52 ± 0.12*
Deferribacteres	0.54 ± 0.05	0.44 ± 0.04	Actinobacteria	0.41 ± 0.16	0.68 ± 0.29
Verrucomicrobia	0.36 ± 0.09	0.19 ± 0.05	[Thermi]	0.52 ± 0.51	0.00 ± 0.00
Tenericutes	0.19 ± 0.08	0.21 ± 0.06	TM7	0.05 ± 0.03	0.19 ± 0.12
Fusobacteria	0.17 ± 0.10	0.05 ± 0.03	Tenericutes	0.05 ± 0.02	0.01 ± 0.00
Deinococcus-Therms	0.07 ± 0.05	0.01 ± 0.01	Acidobacteria	0.03 ± 0.03	0.00 ± 0.00
Cyanobacteria	0.05 ± 0.04	0.01 ± 0.01	Cyanobacteria	0.01 ± 0.01	0.00 ± 0.00
Genus			Genus		
*Lachnospiraceae_NK4A136_group*	3.05 ± 0.26	3.68 ± 0.45	*Akkermansia*	8.00 ± 5.47	3.17 ± 2.60
*Ruminiclostridium*	2.69 ± 0.18	3.48 ± 0.61	*Bacteroides*	4.79 ± 4.36	0.32 ± 0.20
*Ruminiclostridium_9*	2.83 ± 0.18	3.27 ± 0.30	*Oscillospira*	1.64 ± 0.56	0.22 ± 0.07*
*Bacteroides*	3.88 ± 0.75	1.71 ± 0.29*	*Desulfovibrio*	1.62 ± 1.56	0.24 ± 0.11
*Rikenellaceae_RC9_gut_group*	2.49 ± 0.48	2.64 ± 0.32	*Allobaculum*	0.61 ± 0.29	1.03 ± 0.57
*Blautia*	1.96 ± 0.21	2.13 ± 0.20	*Faecalibacterium*	1.18 ± 1.16	0.02 ± 0.02
*Oscillibacter*	1.47 ± 0.15	2.37 ± 0.28*	*Coprococcus*	1.03 ± 0.77	0.03 ± 0.01*
*Roseburia*	1.72 ± 0.24	2.03 ± 0.28	*Ruminococcus*	0.63 ± 0.15	0.19 ± 0.04*
*Alistipes*	2.09 ± 0.14	1.64 ± 0.08*	*Lachnospira*	0.57 ± 0.57	0.01 ± 0.01
*EU457075_g*	1.69 ± 0.21	1.25 ± 0.17	*Sutterella*	0.53 ± 0.51	0.01 ± 0.01

*N* = 7 animals *per* group. * *P* < 0.05 versus YTGCon or ATGCon group, respectively by the Mann–Whitney U test.

**Fig. 6:**
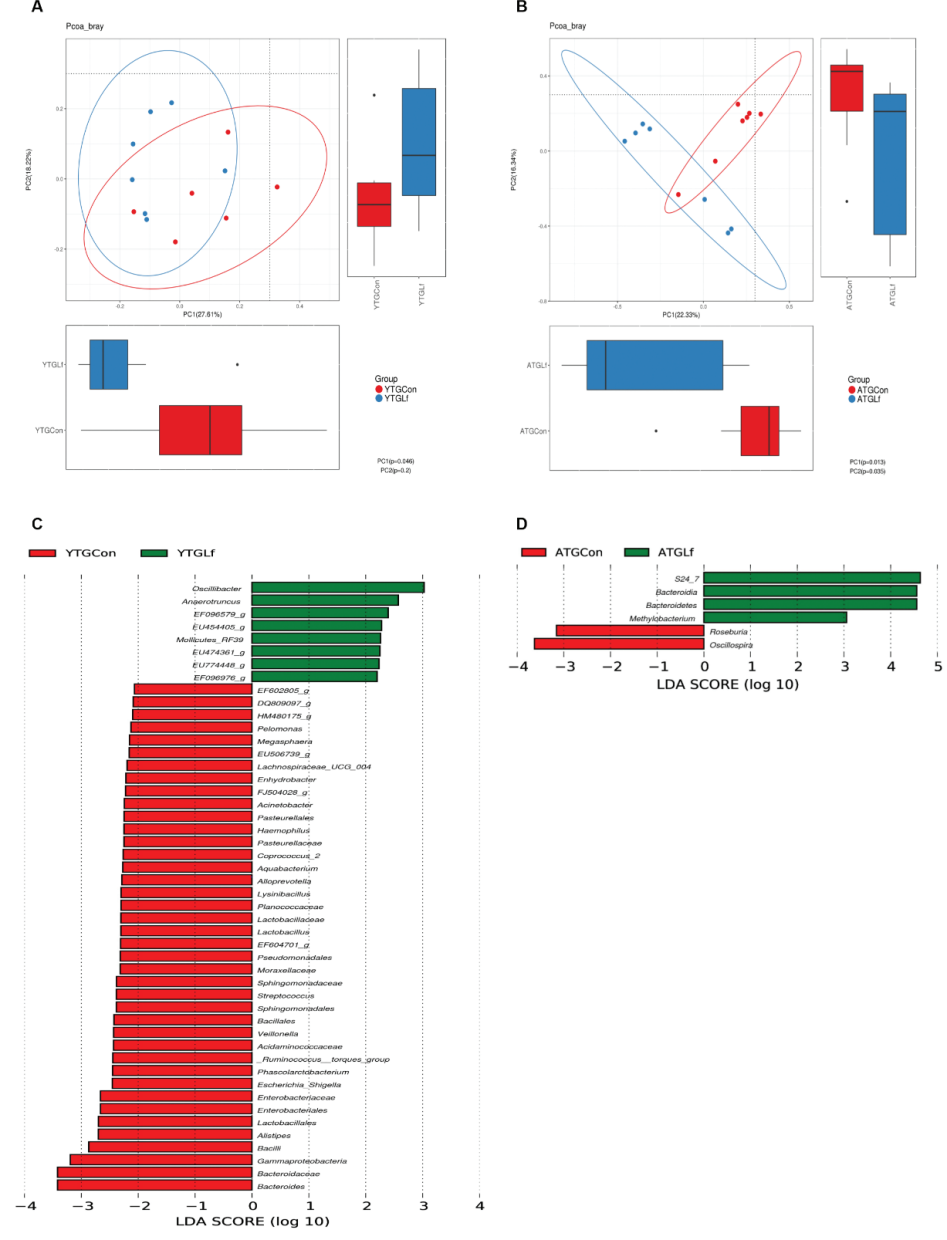
PCoA based on the abundance of OTUs and LEfSe analysis. Principal Coordinate Analysis (PCoA) for gut microbial composition in the young (a) and middle-aged (b) APP/PS1 mice were shown, with the first two principal components plotted to visualize UniFrac distances of cecal samples. Samples from TGCon and TGLf groups were depicted as red and blue, respectively for both the young and middle-aged mice. The bar graph indicated the taxa that discriminate between TGCon and TGLf groups in young (c) and middle-aged (d) APP/PS1 mice, respectively via LEfSe analysis. The statistical test was performed using the LDA effect size method. Only absolute LDA (log10) scores >2.0 were considered statistically significant.

PCoA based on the abundance of OTUs demonstrated differences in the microbial composition (Fig. 6a, b). Specifically, an evidence clustering was identified between the TGCon and TGLf groups for both the young and middle-aged mice. The observation suggested that significant difference in gut microbial community structure existed between the YTGCon and YTGLf, as well as ATGCon and ATGLf groups. The two principal component scores accounted respectively for 27.61% and 18.22% of the total variations for the YTGCon and YTGLf, also 22.33% and 16.34% of the total variations for the ATGCon and ATGLf. We used the LEfSe analysis to compare the statistical differences in microbial communities between TGCon and TGLf groups for both the young and middle-aged mice. As shown in Fig. 6c, there were eight bacterial biomarkers (i.e. *Oscillibacter, Anaerotruncus, EF096579_g, EU454405_g, Mollicutes_RF39, EU474361_g, EU774448_g*, and *EF096976_g*) that were significantly abundant in the YTGLf group in comparison with the YTGCon group. Additionally, four bacterial biomarkers (i.e. *S24_7, Bacteroidia, Bacteroidetes,* and *Methylobacterium*) were significantly greater in the ATGLf group relative to the ATGCon group (Fig. 6d).

## Discussion

We demonstrated that 16 weeks of lactoferrin intervention in both young and middle-aged male APP/PS1 mice might have no effect on cognitive function, consistently, almost no alterations in key markers involved in Aβ, tau pathology, neuro-inflammation, and synaptic plasticity were observed post Lf intervention. However, we demonstrated that Lf intervention could broadly affect gut microbiota profiles, and the effects might be different for the young and middle-aged mice. To be specific, in the young APP/PS1 mice, Lf elevated the α diversity index including ACE and Chao 1, and reduced the relative abundance of the genera *Bacteroides* and *Alistipes* and elevated *Oscillibacter*; in addition, *Oscillibacter, Anaerotruncus, EF096579_g, EU454405_g, Mollicutes_RF39, EU474361_g, EU774448_g*, and *EF096976_g* were specifically abundant post Lf intervention via LEfSe analysis. In the middle-aged APP/PS1 mice, the relative abundance of the phylum *Proteobacteria*, as well as the genera *Oscillospira, Coprococcus,* and *Ruminococcus* was significantly reduced post Lf intervention, additionally, *S24_7*, *Bacteroidia*, *Bacteroidetes,* and *Methylobacterium* were specific via LEfSe analysis post Lf intervention.

Although still inconsistent (9, 10), existing evidence suggest that Lf might exert beneficial effects on AD (7, 8, 30). For example, Carro et al. (30) reported that healthy individuals with low salivary lactoferrin levels had a higher likelihood (more than 77%) of developing AD. Lf administration (orally or intranasally) could improve cognitive function in both AD patients (7), APP/PS1 mice (8) and AβPP (J20) mice (31). Our study was the very first to explore whether Lf intervention on cognition might be age-dependent by using both the young and middle-aged APP/PS1 mice. To our surprise, we observed no protective effects of 16 weeks’ Lf intervention on cognitive function from both the young and middle-aged APP/PS1 mice; we further demonstrated that almost no key markers involved in Aβ metabolism (IDE, ADAM10, BACE1, and cathepsin B), tau phosphorylation (p-tau ser396&404), neuro-inflammation (GFAP and Ibα1), and synaptic plasticity (BDNF and PSD95) were altered post Lf intervention for both young and middle-aged APP/PS1 mice. This is also contradictory to the studies by Abdelhamid et al. (31) and Guo et al. (8). We postulate that the following reasons might explain the absence of beneficial effects of Lf on cognition. First of all, we only set one single dosage of Lf, although previously similar dosages of Lf with 8 weeks intervention from our laboratory had demonstrate beneficial effects of Lf on lipid metabolism (22) in high fat/cholesterol fed mice; we cannot exclude the possibility that the beneficial effects of oral administration of Lf on cognitive function might require higher dosage than the current study or an intervention duration longer than 16 weeks. Secondly, Liu et al. (32) demonstrated that apo-Lf might show better neuroprotective effects on the 1-methyl-4-phenyl-1,2,3,6-tetrahydropyridine (MPTP) induced Parkinson’s disease mouse model than holo-Lf. The Lf used in our study had an iron content of 0.028% (23), which means it is a form of Lf existed between holo-Lf and apo-Lf; therefore, different forms of Lf might result in variable effects on cognitive function. Nevertheless, further studies are still required to explore the effects of Lf intervention on cognition via different AD mouse models with specific emphasis on different forms of Lf with varied dosages.

Existing evidence suggest that Lf could modulate gut microbiota profiles in different species (16–18). For example, in very low birth weight infants, two doses of recombinant human lactoferrin daily intake from day 1 to 28 of life reduced *Enterobacter* and *Klebsiella*, while increasing *Citrobacter* in feces (16). Zhang et al. (18) reported that oral administration of Bovine Lactoferrin-Derived Lactoferricin (Lfcin) B could efficiently maintain gut microbiota homeostasis in the enterohemorrhagic EHEC O157:H7 mouse model. Our study is the very first to demonstrate that 16 weeks’ intervention of Lf might affect gut microbiota profiles differently in young and middle-aged APP/P1 mice.

(1) Effects of Lf on gut microbiota profiles in the young APP/PS1 mice

For the young APP/PS1 mice, Lf could elevate richness of gut bacteria as demonstrated by elevated ACE and Chao1 index compared to the age-matched control group. Meanwhile, at the genus level, the relative abundance of *Bacteroides* and *Alistipes* were reduced while *Oscillibacter* was elevated compared to the control group. *Oscillibacter* has been negatively associated with body mass index or postprandial glucose levels in humans (33). The elevated *Oscillibacter* post Lf intervention in young APP/PS1 mice could suggest that Lf might be beneficial for metabolic parameters, which have been reported by our laboratory previously (22, 23). However, it should be realized our GTT and ITT results did not support the beneficial effects of Lf at least on glucose and insulin metabolism. Elevated *Bacteroides* genus has been reported in nonalcoholic steatohepatitis patients (34). Members of genus *Bacteroides*, that is, *Bacteroides fragil* have been reported to excrete a series of complex neurotoxins that can boost inflammation including surface lipopolysaccharide (LPS) and toxic proteolytic peptides (35). Elevated *Alistipes* has also been reported to be associated with improved gut microbiota composition after dietary intervention for high fat diet (HFD) animals (36). Additionally, Ma et al. (37) reported that genera *Bacteroides* and *Alistipes* were negatively associated with the maintenance of intestine redox in tea polyphenols treated HFD fed mice. The reduction of *Bacteroides* and *Alistipes* post Lf in young mice might also suggest these specific bacteria play functional roles in the oxidative stress and anti-inflammatory response. The LEfSe analysis demonstrated that *Oscillibacter, Anaerotruncus, EF096579_g, EU454405_g, Mollicutes_RF39, EU474361_g, EU774448_g, and EF096976_g* were specifically abundant in young APP/PS1 mice post Lf intervention. The functional roles of these abundant bacteria post Lf intervention require further exploration.

(2) Effects of Lf on gut microbiota profiles in the middle-aged APP/PS1 mice

Increased abundance of phylum *Proteobacteria* has been proposed to be a potential diagnostic signature of gut dysbiosis (38). The genera *Oscillospira* is generally considered as an anti-inflammatory bacteria, and is positively associated with leanness and health (39). The genus *Coprococcus* has usually been reported to be beneficial for maintaining intestinal stability (40). Elevated abundance of genera *Ruminococcus* is associated with irritable bowel syndrome (41), and is usually implicated in negative health outcomes including AD (42). The reduction in phylum *Proteobacteria*, and genera *Ruminococcus* post Lf intervention suggest that Lf might improve gut microbiota profiles in the middle-aged APP/PS1 mice, while the functional roles of reduction in *Oscillospira* and *Coprococcus* post Lf intervention require further exploration. The LEfSe analysis demonstrated that *S24-7*, *Bacteroidia*, *Bacteroidetes,* and *Methylobacterium* were specifically abundant post Lf treatment in the middle-aged APP/PS1 mice. Similar to those in young APP/PS1 mice, the functional roles of these abundant bacteria post Lf intervention in the middle-aged APP/PS1 mice also require further exploration.

Taken together, although dietary lactoferrin has no effect on the cognitive ability of both two age groups, it could elevate gut bacteria richness of young group and increase antioxidative stress and anti-inflammatory gut microbiota profiles for both young and middle-aged mice. Therefore, dietary lactoferrin might play a potential beneficial role in maintaining intestinal homeostasis and preventing related diseases. However, the specific mechanism of how these changes affect the host needs to be further explored in the future by bacteria transplantation etc. We think this is also one of the limitations of the present study.

## Conclusions

In summary, we demonstrated that 16 weeks Lf intervention had no effect on cognitive function, and key AD related markers including Aβ, tau pathology, neuro-inflammation, and synaptic plasticity from both the young and middle-aged APP/PS1 mice; while Lf differentially affected gut microbiota profiles. Our findings could indicate that dietary Lf might be beneficial for gut microbiota homeostasis although it might have no effects on cognition.
